# Distinguishing COVID-19 From Influenza Pneumonia in the Early Stage Through CT Imaging and Clinical Features

**DOI:** 10.3389/fmicb.2022.847836

**Published:** 2022-05-06

**Authors:** Zhiqi Yang, Daiying Lin, Xiaofeng Chen, Jinming Qiu, Shengkai Li, Ruibin Huang, Zhijian Yang, Hongfu Sun, Yuting Liao, Jianning Xiao, Yanyan Tang, Xiangguang Chen, Sheng Zhang, Zhuozhi Dai

**Affiliations:** ^1^Department of Radiology, Meizhou People’s Hospital, Meizhou, China; ^2^Department of Radiology, Shantou Central Hospital, Shantou, China; ^3^Department of Radiology, Second Affiliated Hospital, Shantou University Medical College, Shantou, China; ^4^Department of Radiology, Huizhou Municipal Central Hospital, Huizhou, China; ^5^Department of Radiology, First Affiliated Hospital, Shantou University Medical College, Shantou, China; ^6^Department of Radiology, Yongzhou People’s Hospital, Yongzhou, China; ^7^The University of Queensland School of Information Technology and Electrical Engineering, Brisbane, QLD, Australia; ^8^GE Healthcare, Guangzhou, China

**Keywords:** COVID-19, influenza pneumonia, CT features, clinical features, differential diagnosis

## Abstract

**Background:**

Both coronavirus disease 2019 (COVID-19) and influenza pneumonia are highly contagious and present with similar symptoms. We aimed to identify differences in CT imaging and clinical features between COVID-19 and influenza pneumonia in the early stage and to identify the most valuable features in the differential diagnosis.

**Methods:**

Seventy-three patients with COVID-19 confirmed by real-time reverse transcription-polymerase chain reaction (RT-PCR) and 48 patients with influenza pneumonia confirmed by direct/indirect immunofluorescence antibody staining or RT-PCR were retrospectively reviewed. Clinical data including course of disease, age, sex, body temperature, clinical symptoms, total white blood cell (WBC) count, lymphocyte count, lymphocyte ratio, neutrophil count, neutrophil ratio, and C-reactive protein, as well as 22 qualitative and 25 numerical imaging features from non-contrast-enhanced chest CT images were obtained and compared between the COVID-19 and influenza pneumonia groups. Correlation tests between feature metrics and diagnosis outcomes were assessed. The diagnostic performance of each feature in differentiating COVID-19 from influenza pneumonia was also evaluated.

**Results:**

Seventy-three COVID-19 patients including 41 male and 32 female with mean age of 41.9 ± 14.1 and 48 influenza pneumonia patients including 30 male and 18 female with mean age of 40.4 ± 27.3 were reviewed. Temperature, WBC count, crazy paving pattern, pure GGO in peripheral area, pure GGO, lesion sizes (1–3 cm), emphysema, and pleural traction were significantly independent associated with COVID-19. The AUC of clinical-based model on the combination of temperature and WBC count is 0.880 (95% CI: 0.819–0.940). The AUC of radiological-based model on the combination of crazy paving pattern, pure GGO in peripheral area, pure GGO, lesion sizes (1–3 cm), emphysema, and pleural traction is 0.957 (95% CI: 0.924–0.989). The AUC of combined model based on the combination of clinical and radiological is 0.991 (95% CI: 0.980–0.999).

**Conclusion:**

COVID-19 can be distinguished from influenza pneumonia based on CT imaging and clinical features, with the highest AUC of 0.991, of which crazy-paving pattern and WBC count play most important role in the differential diagnosis.

## Introduction

The coronavirus disease 2019 (COVID-19) pandemic caused by the novel coronavirus SARS-CoV-2 is a global crisis and has a significant impact on global public health and social systems (Bai and Tao, 2021; Caruso et al., 2021b; Chotpitayasunondh et al., 2021). The most typical clinical symptoms of COVID-19 are fever, cough, fatigue, and myalgia. The most common results of laboratory tests are leukopenia and lymphopenia (Chen et al., 2020; Henry and Vikse, 2020; Singh et al., 2021). However, these manifestations of COVID-19 usually overlap with those of influenza pneumonias (Pan et al., 2020; Wang et al., 2020).

CT examination plays a significant role in the differential diagnosis, the monitoring of disease progression, evaluating the treatment effectiveness and patient outcomes. The most typical radiological finding is ground-glass opacity (GGO) with or without consolidation, especially pure GGO (Wang et al., 2020; Booz et al., 2021), in the subpleural region, located unilaterally or bilaterally in the lower lobes (Pan et al., 2020). The lesions can develop one or more lobes, with a slight preference for the lower right lobe (Han et al., 2020). In addition, a diversity of interesting CT features including crazy paving pattern and airway changes were found with further analysis of increasing cases (Ye et al., 2020). However, these CT imaging findings are also similar to those of influenza pneumonia (Wang et al., 2014; Shi et al., 2020). Therefore, the discrimination between COVID-19 and influenza is critical in clinical practice. Accurate imaging and clinical features recognition can aid in the early diagnosis of COVID-19 and thus prevent spreading and speed up treatment.

In our previous study (Chen et al., 2020), we demonstrated that based on CT imaging and clinical manifestations alone, pneumonia patients with and without COVID-19 can be distinguished. Harrison et al. examined the performance of seven radiologists in differentiating COVID-19 from viral pneumonia on chest CT results and found an average sensitivity of 80% and a specificity of 84% (Bai et al., 2020). However, we realized that approximately 44% of the viral pneumonia cases were human rhinovirus, and influenza pneumonia accounted for only approximately 15% of the cases in the study by Harrison et al. In this study, we aimed to identify differences in CT imaging and clinical features between COVID-19 and influenza pneumonia in the early stage and to identify the most valuable features in distinguishing COVID-19 from influenza pneumonia, based on multicenter data.

## Materials and Methods

### Patients

Ethical approval by the institutional review boards was obtained for this retrospective analysis, with the requirement for informed consent waived. From January 1 to February 15 2020, 73 consecutive patients confirmed with SARS-CoV-2 infection by real-time reverse transcription-polymerase chain reaction (RT-PCR) from five independent hospitals in four Chinese cities were enrolled in this study. The inclusion criteria of COVID-19 patients who underwent chest CT were positive for RT-PCR tests without vaccines. No patients were excluded. All of patients, 64 patients had mild COVID-19 pneumonia and nine patients had moderate COVID-19 pneumonia. Among them, 41 patients were men (mean age: 41.4 years; range: 16–69 years), and 32 were women (mean age: 42.6 years; range: 3–66 years). In our prior studies (Chen et al., 2020; Yang et al., 2021), we reported on 63 mild COVID-19 pneumonia and seven moderate COVID-19 pneumonia included in the current study for the differentiating COVID-19 from non-COVID-19, as well as 64 mild COVID-19 patients and nine moderate COVID-19 patients for the evaluating the three atypical presentations of COVID-19. However, no influenza pneumonia patients in this study have been reported or published before. The imaging differences between COVID-19 and influenza pneumonia have not been reported.

**Figure 1:**
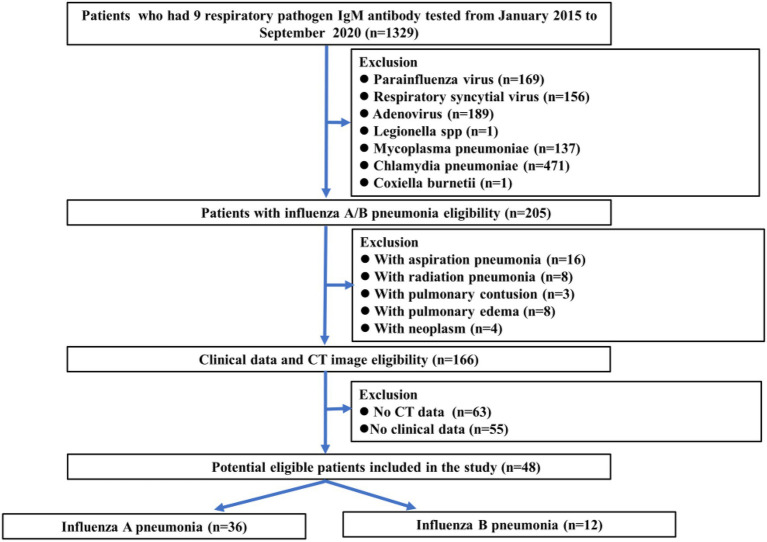
Flowchart showing influenza pneumonia patient selection along with the inclusion and exclusion criteria.

In addition, from January 1 2015, to September 30 2019, a total of 205 consecutive patients with influenza pneumonia from Shantou and Meizhou cities were reviewed. The inclusion criteria of influenza pneumonia patients who underwent chest CT were positive for direct/indirect immunofluorescence antibody staining or RT-PCR tests. The exclusion criteria of influenza pneumonia were as follows: (1) with aspiration pneumonia (*n* = 16); (2) with radiation pneumonia (*n* = 8); (3) with pulmonary contusion (*n* = 3); (4) with pulmonary edema (*n* = 8); (5) with neoplasm (*n* = 4); (6) CT imaging-negative patients (*n* = 63); and (7) no clinical data (*n* = 55; Figure 1). Finally, 48 influenza pneumonia patients (mean age: 40.4 years, range: 0.1–83 years) were enrolled as controls, including 30 men (mean age: 40.1 years; range: 0.1–72 years) and 18 women (mean age: 40.8 years; range: 0.1–83 years).

### Image and Clinical Data Collection

Non-contrast-enhanced chest CT imaging data were obtained from multiple hospitals with varied CT systems, including GE CT Discovery 750 HD (General Electric, United States), SCENARIA 64 CT (Hitachi Medical, Japan), PHILIPS Ingenuity CT (PHILIPS, Netherlands), and Siemens SOMATOM Definition AS (Siemens, Germany) systems. All images were reconstructed into 1-mm slices with a slice interval of 0.8 mm, a lung window width of 1,500 HU, and a window level of −500 HU. The detailed acquisition parameters are summarized in the Supplementary Material (Supplementary Table E1).

Baseline clinical data including course of disease, age, sex, body temperature, clinical symptoms (including cough, fatigue, sore throat, stuffy, and runny nose), total white blood cell (WBC) count, lymphocyte count, lymphocyte ratio, neutrophil count, neutrophil ratio and C-reactive protein (CRP) level were collected. According to the normal range used at individual hospitals, the threshold values for WBC count, lymphocyte count, lymphocyte ratio, neutrophil count, neutrophil ratio, and CRP level were set to 3.5–9.5 × 10^9^/L, 1.1–3.2 × 10^9^/L, 20.0%–50.0%, 1.8–6.3 × 10^9^/L, 40.0%–75.0%, and 0.0–6.0 mg/L, respectively.

### CT Image Analysis

**Figure 2:**
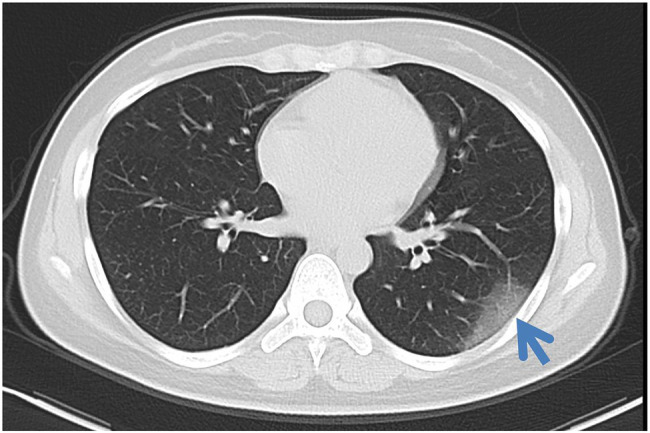
Axial non-contrast-enhanced CT image from a 26-year-old female patient with COVID-19. Pure ground-glass opacities were observed in the peripheral area in the left lower lobe. The maximum diameter of the lesion was 4.5 cm. The left lower lobe score was 1 because the lung parenchyma was less than 25%.

A total of 22 qualitative and 25 numerical imaging features were extracted for analysis. The descriptions of the CT qualitative and numerical imaging features are listed in the Supplementary Material (Supplementary Tables E2, E3). For the extraction of CT qualitative and numerical imaging features, two senior radiologists (ZY and XC, more than 15 years of experience) reached a consensus and were blinded to the clinical and laboratory findings. The combination of GGO and consolidation was defined as mixed ground-glass opacity. The vessel associated with lesions enlarged in CT images was defined as offending vessel augmentation in lesions. Lesions in the outer third of the lung were defined as peripheral lesions, and lesions in the inner two-thirds of the lung were defined as central lesions. The classification of the lesion size was based on a previous study (Das et al., 2015). The progression of lesions within each lung lobe was evaluated by scoring each lobe from 0 to 4 (Chung et al., 2020), corresponding to normal, 1%–25% infection, 26%–50% infection, 51%–75% infection, and more than 75% infection, respectively. The scores were combined for all five lobes to provide a total score ranging from 0 to 20. Figure 2 is one example of the evaluation of chest CT images.

### Statistical Analysis

The CT imaging and clinical features were compared between the COVID-19 and influenza pneumonia groups by using the Chi-square test (for nominal variables), the Kruskal–Wallis *H* test (for ordinal variables), or the Student’s *t* test (for continuous variables). The features with a significant difference between the two groups were extracted. Spearman or Kendall correlation tests between feature metrics and diagnosis outcomes (i.e., 1 for COVID-19 and 0 for influenza pneumonia) were assessed for each extracted feature. The diagnostic performance of clinical and CT features in differentiating COVID-19 from influenza pneumonia was evaluated with univariate and multivariate analyses. Additionally, the corresponding area under the curve (AUC), accuracy, specificity, sensitivity, and threshold were calculated. All statistical analyses for this study were performed with R (version 3.6.4).^1^ A two-tailed value of *p* < 0.05 indicated statistical significance.

## Results

### Clinical Features Comparison Between Groups

The course of disease was 2.66 ± 2.62 days for COVID-19 and 2.19 ± 2.10 days for influenza pneumonia. The clinical features of COVID-19 and influenza pneumonia patients are shown in Table 1. Compared to COVID-19 patients, influenza pneumonia patients had higher temperatures (*p* < 0.001), WBC counts (*p* < 0.001), neutrophil counts (*p* < 0.001), neutrophil rates (*p* = 0.017), and CRP levels (*p* = 0.033), and lower lymphocyte rates (*p* = 0.005), which were also confirmed by multiple tests.

**Table 1:** Clinical features of COVID-19 and influenza pneumonia patients.

Clinical features	COVID-19 (*n* = 73)	Influenza (*n* = 48)	*p*	Adjust value of *p*
*Sex*				
Male^#^	41 (56.16%)	30 (62.50%)	0.489^a^	NA
Female^#^	32 (43.84%)	18 (37.50%)		
Age (years)	41.92 ± 14.11	40.38 ± 27.31	0.720^b^	0.720
Course of disease (days)	2.66 ± 2.62	2.19 ± 2.10	0.299^b^	0.336
Temperature (°C)	37.17 ± 0.85	38.23 ± 1.25	<0.001^b^^,^^*^	0.003^*^
*Symptoms*				
Cough^#^	50 (68.49%)	37 (77.08%)	0.304^a^	NA
Fatigue^#^	22 (30.14%)	16 (33.33%)	0.711^a^	NA
Sore throat^#^	9 (12.33%)	5 (10.42%)	0.748^a^	NA
Stuffy^#^	2 (2.74%)	5 (10.42%)	0.170^a^	NA
Runny nose^#^	3 (4.11%)	7 (14.58%)	0.087^a^	NA
WBC count (×10^9^/L)	5.36 ± 2.35	9.67 ± 5.32	<0.001^b^^,^^*^	0.003^*^
Lymphocyte count (×10^9^/L)	1.33 ± 0.85	1.66 ± 1.63	0.196^b^	0.252
Lymphocyte ratio (%)	25.46 ± 11.45	18.92 ± 13.76	0.005^b^^,^^*^	0.011^*^
Neutrophil count (×10^9^/L)	3.53 ± 2.13	7.11 ± 4.65	<0.001^b^^*^	0.003^*^
Neutrophil ratio (%)	64.35 ± 14.35	71.28 ± 17.06	0.017^b^^,^^*^	0.031^*^
C-reactive protein (mg/L)	22.46 ± 31.08	38.79 ± 45.56	0.033^b^^,^^*^	0.049^*^

Adjust value of *p*: multiple test. NA, not available and WBC, white blood cell.

* Data with statistical significance.

# Results are measurements with corresponding ratio in parentheses, and the remainder results are mean value with SD.

a Chi square test.

b Student’s *t* test.

### Imaging Features Comparison Between Groups

The CT numerical and qualitative imaging features between COVID-19 and influenza pneumonia are shown in Supplementary Tables E4, E5 in the Supplementary Materials, respectively. Those features with significant differences are presented in Table 2. For all numerical imaging features, COVID-19 patients had a greater total number of pure GGOs (*p* = 0.01), the total number of pure GGOs in the peripheral area (*p* = 0.003), the total number of mixed GGO in the peripheral area (*p* = 0.016), and the total number of lesions in the peripheral area (*p* = 0.003). However, COVID-19 patients had a lower total number of consolidations (*p* = 0.018) and total scores of the left lung (*p* = 0.032). Compared to influenza pneumonia patients, more lesions were between 1 and 3 cm (*p* = 0.005) in COVID-19 patients.

**Table 2:** CT imaging features with significant differences between COVID-19 and influenza pneumonia patients.

Numerical imaging features	COVID-19 (*n* = 73)	Influenza (*n* = 48)	*p*	Adjust value of *p*
*Number of pure GGO*				
Total	6.78 ± 11.28	2.75 ± 5.33	0.010^b^^,^^*^	0.020^*^
Peripheral area	4.81 ± 7.15	1.92 ± 3.16	0.003^b^^,^^*^	0.012^*^
Number of mixed GGO in peripheral area	4.60 ± 6.92	2.15 ± 4.12	0.016^b^^,^^*^	0.021^*^
Number of consolidations	0.60 ± 1.65	1.60 ± 2.52	0.018^b^^,^^*^	0.021^*^
Total number of lesions in peripheral area	10.74 ± 13.69	5.15 ± 6.63	0.003^b^^,^^*^	0.012^*^
Lesion sizes (1–3 cm)	8.29 ± 14·24	3.21 ± 4.·19	0.005^b^^,^^*^	0.013^*^
*Total scores of involved lung zones*				
Left lung	2.15 ± 1.86	3.10 ± 2.62	0.032^b^^,^^*^	0.032^*^
Bilateral lower lobes	2.59 ± 2.18	3.69 ± 2.52	0.015^b^^,^^*^	0.021^*^
**Qualitative imaging features**	COVID-19 (*n* = 73)	Influenza (*n* = 48)	*p*	Adjust value of *p*
Pure GGO^#^			0.008^a^^,^^*^	NA
Negative	18 (24.7%)	23 (47.9%)		
Positive	55 (75.3%)	25 (25.1%)		
Pure GGO in peripheral area^#^			0.004^a^^,^^*^	NA
Negative	19 (26.0%)	25 (52.1%)		
Positive	54 (74.0%)	23 (47.9%)		
Mixed GGO^#^			0.020^a^^,^^*^	NA
Negative	16 (21.9%)	20 (41.7%)		
Positive	57 (78.1%)	28 (58.3%)		
Mixed GGO in peripheral area^#^			<0.001^a^^,^^*^	NA
Negative	18 (24.7%)	27 (56.3%)		
Positive	55 (75.3%)	21 (43.7%)		
Consolidation^#^			<0.001^a^^,^^*^	NA
Negative	56 (76.7%)	21 (43.8%)		
Positive	17 (23.3%)	27 (56.2%)		
Interlobular septal thickening^#^			0.037^a^^,^^*^	NA
Negative	33 (45.21%)	31 (64.58%)		
Positive	40 (54.79%)	17 (35.42%)		
Crazy paving pattern^#^			<0.001^a^^,^^*^	NA
Negative	35 (47.95%)	41 (85.42%)		
Positive	38 (52.05%)	7 (14.58%)		
Offending vessel augmentation in lesions^#^			0.021^a^^,^^*^	NA
Negative	20 (27.40%)	23 (47.92%)		
Positive	53 (72.60%)	25 (52.08%)		
Pleural traction^#^			0.007^a^^,^^*^	NA
Negative	38 (52.05%)	13 (27.08%)		
Positive	35 (47.95%)	35 (72.92%)		
Emphysema^#^			0.045^a^^,^^*^	NA
Negative	67 (91.78%)	38 (79.17%)		
Positive	6 (8.22%)	10 (20.83%)		
Pleural effusions^#^			<0.001^a^^,^^*^	NA
Negative	73 (100.0%)	38 (79.17%)		
Positive	0 (0.00%)	10 (20.83%)		
Lymphadenopathy^#^			0.047^a^^,^^*^	NA
Negative	73 (100.0%)	44 (91.67%)		
Positive	0 (0.00%)	4 (8.33%)		

Adjust value of *p*: multiple test. NA, not available and GGO, ground-glass opacity.

* Data with statistical significance.

# Results are measurements with corresponding ratio in parentheses, and the remainder results are mean value with SD.

a Chi-square test.

b Student’s *t* test.

For all qualitative imaging features, most COVID patients presented a higher positive rate of interlobular septal thickening (54.79%), crazy-paving pattern (52.05%), and offending vessel augmentation in lesions (72.60%), and a lower positive rate of pleural traction (47.95%), emphysema (8.22%), pleural effusions (0.00%), and lymphadenopathy (0.00%). Compared to the COVID-19 patients, the decreased positive rate of interlobular septal thickening (35.42%), crazy-paving pattern (14.58%), offending vessel augmentation in lesions (52.08%), as well as an increased positive rate of pleural traction (72.92%), emphysema (20.83%), pleural effusions (20.83%), and lymphadenopathy (8.83%) are more pronounced in influenza virus infection patients (all *p* < 0.05).

### Correlation Analysis and Diagnostic Performance for Clinical Features

**Figure 3:**
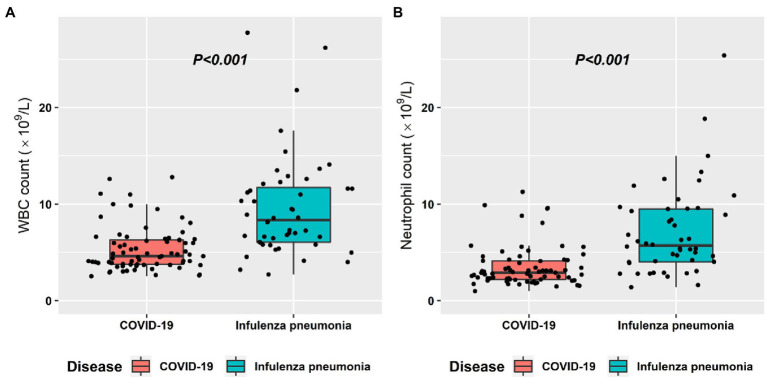
Box plot graphs revealing statistically significant differences in both the white blood cell (WBC) counts **(A)** and the neutrophil count **(B)** between COVID-19 and influenza pneumonia patients. Most patients with both diseases had normal WBC counts and neutrophil counts; however, the overall values in influenza pneumonia were higher than those in COVID-19 (*p* < 0.001).

The correlation analysis and diagnostic performance of clinical features in distinguishing COVID-19 from influenza pneumonia are shown in Table 3. The diagnostic outcomes correlated significantly with the WBC count (Spearman’s *r* correlation, *r* = −0.526, *p* < 0.001) and neutrophil count (*r* = −0.500, *p* < 0.001). The lymphocyte rate and temperature had a weaker correlation with distinguishing COVID-19 from influenza pneumonia, with *r* = 0.310 (*p* < 0.001) and *r* = −0.433 (*p* < 0.001), respectively. However, few correlations were found for C-reactive protein and neutrophil ratio in the differential diagnosis. The WBC count yielded a maximum AUC of 0.811 (95% CI: 0.731–0.890), followed by the neutrophil count with an AUC of 0.795 (95% CI: 0.711–0.879). The distribution of WBC count and neutrophil count in both groups is shown in Figure 3. In the multivariable analysis, WBC count (OR = 0.47, *p* = 0.043) and temperature (OR = 0.34, *p* < 0.001) were significantly independent associated with COVID-19. The AUC of clinical-based model on the combination of WBC count and temperature is 0.880 (95% CI: 0.819–0.939).

**Table 3:** Correlation analysis and diagnostic performance of clinical features in distinguishing COVID-19 from influenza pneumonia.

Clinical features	Correlation analysis		ROC analysis					
	*r*	*p*	AUC	95% CI	Accuracy	Specificity	Sensitivity	Threshold
Lymphocyte ratio	0.310^a^	<0.001	0.683	0.581–0.785	0.686	0.616	0.792	23.65
C-Reactive protein	–0.204^a^	0.025	0.620	0.517–0.724	0.661	0.822	0.417	34.82
Neutrophil ratio	–0.264^a^	0.003	0.656	0.552–0.760	0.669	0.658	0.688	65.78
Temperature	–0.433^a^	<0.001	0.755	0.663–0.847	0.744	0.890	0.521	38.15
Neutrophil count	–0.500^a^	<0.001	0.795	0.711–0.879	0.769	0.822	0.688	4.610
WBC count	–0.526^a^	<0.001	0.811	0.731–0.890	0.760	0.781	0.729	6.435
Clinical-based model	NA	NA	0.880	0.819–0.939	0.793	0.875	0.739	0.972

Clinical-based model indicate the predicted model based on the combination of WBC count and temperature. NA, not available; CI, confidence interval; and WBC, white blood cell.

a *r* and corresponding value of *p* are computed by Spearman’s correlation test.

### Correlation Analysis and Diagnostic Performance for CT Features

**Figure 4:**
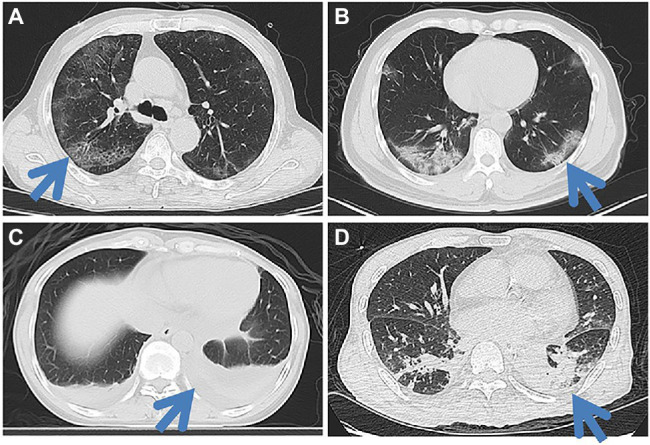
Typical CT imaging features in both COVID-19 patients **(A,B)** and influenza pneumonia patients **(C,D)**. A 65-year-old man with COVID-19 **(A)** shows the crazy-paving pattern sign in the posterior segment of the right upper lobe along with bilateral peripheral multifocal ground-glass opacities (GGOs). A 46-year-old man with a COVID-19 **(B)** shows multifocal mixed GGOs in the lower lobe of both lungs, mainly in the peripheral. A 44-year-old female with influenza pneumonia shows lower lobe atelectasis in the posterior basal segment of both lungs, along with bilateral pleural effusions. A 60-year-old man with influenza pneumonia shows local consolidations in the posterior segment and lateral basal segment of both lower lobes.

The correlation analysis and diagnostic performance of CT features in distinguishing COVID-19 from influenza pneumonia are shown in Table 4. Crazy-paving pattern was more common in COVID-19 than in influenza pneumonia, while consolidation and pleural effusions were more common in influenza pneumonia than in COVID-19. In the multivariable analysis, crazy paving pattern (OR = 796, *p* = 0.007), pure GGO in peripheral area (OR = 306, *p* = 0.014), pure GGO (OR < 0.001, *p* = 0.012), lesion sizes (1–3 cm) (OR = 1.68, *p* = 0.015), emphysema (OR = 0.03, *p* = 0.044), and pleural traction (OR = 0.10, *p* = 0.034) were significantly independent associated with COVID-19. The AUC of radiological-based model on the combination of crazy paving pattern, pure GGO in peripheral area, pure GGO, lesion sizes (1–3 cm), emphysema, and pleural traction is 0.957 (95% CI: 0.924–0.989). The AUC of combined model based on the combination of clinical and radiological features is 0.991 (95% CI: 0.980–0.999). The typical CT imaging features of both diseases are illustrated in Figure 4. In addition, literature comparison between COVID-19 and influenza is illustrated in Supplementary Table E6 in the Supplementary Materials.

**Table 4:** Correlation analysis and diagnostic performance of CT features in distinguishing COVID-19 from influenza pneumonia.

CT features	Correlation analysis		ROC analysis					
	*r*	*p*	AUC	95% CI	Accuracy	Specificity	Sensitivity	Threshold
Crazy paving pattern	0.379^b^	<0.001	0.687	0.611–0.764	0.653	0.521	0.854	0.426
Mixed GGO in peripheral area	0.320^b^	<0.001	0.658	0.571–0.745	0.678	0.753	0.563	0.478
Pure GGO in peripheral area	0.265^b^	0.004	0.630	0.543–0.718	0.653	0.740	0.521	0.481
Total number of lesions in peripheral area	0.248^a^	0.006	0.646	0.547–0.745	0.652	0.644	0.667	4.499
Pure GGO	0.240^b^	0.008	0.616	0.529–0.703	0.645	0.753	0.479	0.483
Lesion sizes(1–3 cm)	0.220^a^	0.015	0.629	0.530–0.728	0.603	0.534	0.708	3.498
Mixed GGO	0.211^b^	0.021	0.599	0.514–0.684	0.636	0.781	0.417	0.486
Offending vessel augmentation in lesions	0.210^b^	0.022	0.603	0.515–0.691	0.628	0.726	0.479	0.484
Interlobular septal thickening	0.190^b^	0.037	0.597	0.508–0.686	0.586	0.548	0.646	0.478
Total scores of left lung	-0.154^a^	0.092	0.590	0.484–0.695	0.636	0.795	0.396	3.503
Emphysema	-0.182^b^	0.046	0.563	0.497–0.629	0.636	0.918	0.208	0.502
Total scores of bilateral upper lobes	-0.210^a^	0.021	0.622	0.521–0.724	0.620	0.699	0.500	3.504
Lymphadenopathy	-0.228^b^	0.012	0.541	0.502–0.581	0.636	1.000	0.083	0.076
Pleural traction	-0.247^b^	0.007	0.625	0.539–0.711	0.603	0.521	0.729	0.534
Consolidation	-0.335^b^	<0.001	0.665	0.579–0.751	0.686	0.767	0.563	0.521
Pleural effusions	-0.370^b^	<0.001	0.604	0.546–0.662	0.686	1.000	0.208	0.075
Radiological-based model	NA	NA	0.956	0.923–0.989	0.909	0.875	0.931	0.160

Radiological-based model indicates the predicted model based on the combination of crazy paving pattern, pure GGO in peripheral area, pure GGO, lesion sizes (1–3 cm), emphysema, and pleural traction. NA, not available; CI, confidence interval; and GGO, ground-glass opacity.

a *r* and corresponding value of *p* are computed by Spearman’s correlation test.

b *r* and corresponding value of *p* are computed by Kendall correlation test.

## Discussion

In this study, we compared CT imaging and clinical manifestations between COVID-19 and influenza pneumonia and identified the most valuable features for differential diagnosis. Our study showed that the WBC count had the highest correlation (*r* = −0.526, *p* < 0.001), followed by the neutrophil count (*r* = −0.500, *p* < 0.001). Four CT imaging features were identified as the most significant for differential diagnosis, including crazy-paving pattern, mixed GGO in the peripheral area, pleural effusions, and consolidation. The AUC of combined model based on the combination of clinical and radiological features yielded a maximum AUC of 0.991 (95% CI: 0.980–0.999) for differentiating COVID from influenza pneumonia.

In previous studies, GGOs in the periphery have become a recognized indicator of COVID-19 in the early stage (Kanne, 2020; Shi et al., 2020). In line with previous studies, we found that in the early stage of COVID-19, approximately 78% of patients had mixed GGOs. However, this feature only ranked third among the 25 extracted features for distinguishing COVID-19 from influenza. The crazy-paving pattern, which has also been reported in previous studies (Pan et al., 2020; Xu et al., 2020b), achieved the highest AUC for differential diagnosis. These two features were also reported in other coronavirus diseases, such as severe acute respiratory syndrome (SARS) and Middle East respiratory syndrome (MERS; Chan et al., 2004; Ajlan et al., 2014). The pathology of COVID-19 was confirmed to greatly resemble those of SARS and MERS (Guarner, 2020; Xu et al., 2020a). Tian et al. (2020) reported that the lungs of COVID-19 patients exhibited edema, proteinaceous exudate, focal reactive hyperplasia of pneumocytes with patchy inflammatory cellular infiltration, and multinucleated giant cells, which can cause the thickening of interlobular septa and represented a crazy paving pattern. Consistent with previous reports (Ye et al., 2020), pleural effusions are very rare in COVID-19 patients, ranking second among CT imaging features for differential diagnosis.

Compared to the COVID-19, the most common imaging findings of influenza are consolidation and bronchial wall thickening (Franquet, 2011; Koo et al., 2018). In this study, we found that over 56% of influenza patients had positive consolidation, while the positive rate was only 23% for COVID-19 patients in the early stage. The positive rate is significantly different, with a value of *p* less than 0.001. However, Bernheim et al. found that in a long time after onset, more consolidation was present in COVID-19 patients (Bernheim et al., 2020), which was also confirmed by Shi et al. (2020). Therefore, in the follow-up of the disease, the difference in this feature between the two diseases may be weakened. Bronchial wall thickening was suggested to be not significantly different between influenza and COVID-19 pneumonia (*p* = 0.715), which indicated that both diseases could affect airway walls.

In contrast to RT-PCR and serological antibody tests for the diagnosis of COVID-19, which may need more time and have a false-negative rate, imaging and clinical findings have the advantage of reflecting the disease earlier. A recent study published by Lin et al. also found that the imaging features were different between these two diseases (Lin et al., 2020). However, the diagnostic performance of each feature has not been evaluated previously. Moreover, because every individual feature has limited diagnostic efficacy, the prediction model combining multiple parameters becomes a viable alternative. By incorporating all the variables into the prediction model, the overall predictive ability was strong, with a maximum AUC of 0.991.

There are several limitations in this study. First, in order to evaluate the differential diagnosis in the early stage, we only compared only the initial CT scanning of both COVID-19 and influenza pneumonia. Since the CT manifestations change over the course of the disease (Wang et al., 2020), our results may be biased at different time windows (Caruso et al., 2021a). Second, there may be some inherent deviations in the multicenter retrospective design (Sica, 2006), since the scanning protocols are slightly diverse among different hospitals. Finally, although the preliminary results are promising, all the data come from five research institutions in the Guangdong and Hunan Provinces. The included data may be potentially biased. In addition, the sample size selected in both groups is relatively small. Further validation on a larger dataset in different regions is needed to determine the potential of these features for distinguishing COVID-19 from influenza pneumonia. After validation, further diagnostic models may be created based on these features.

## Conclusion

COVID-19 can be distinguished from influenza pneumonia based on CT imaging and clinical features, with the highest AUC of 0.991, of which crazy-paving pattern and WBC count play most important role in the differential diagnosis.

## Data Availability Statement

The data cohorts used and/or analyzed during the present study are available from the corresponding author on reasonable request.

## Ethics Statement

The studies involving human participants were reviewed and approved by Meizhou People’s Hospital. Written informed consent for participation was not provided by the participants’ legal guardians/next of kin because: Written informed consent was waived in light of the urgent need to collect data.

## Author Contributions

ZqY was involved in the data curation and writing—original draft preparation. DL and XoC contributed to the data curation, writing—reviewing and editing, and methodology. JQ, SL, RH, and ZjY were involved in the data curation. HS contributed to the resources. YL contributed to the methodology and visualization. JX and YT helped in the data curation. XnC and SZ were involved in the conceptualization, investigation, and supervision. ZD contributed to the conceptualization, investigation, writing—reviewing and editing, and supervision. All authors contributed to the article and approved the submitted version.

## Funding

This work was supported by grants from the Natural Science Foundation of Guangdong Province (grant number 2018A030307057) to ZD; the Department of Education of Guangdong Province (grant number 2020KZDZX1085) to ZD; and the Meizhou Social Science and Technology Planning Development Project (grant number 2020B103) to ZqY.

## Conflict of Interest

The authors declare that the research was conducted in the absence of any commercial or financial relationships that could be construed as a potential conflict of interest.

## Publisher’s Note

All claims expressed in this article are solely those of the authors and do not necessarily represent those of their affiliated organizations, or those of the publisher, the editors and the reviewers. Any product that may be evaluated in this article, or claim that may be made by its manufacturer, is not guaranteed or endorsed by the publisher.

## Supplementary Material

The Supplementary Material for this article can be found online at: https://www.frontiersin.org/articles/10.3389/fmicb.2022.847836/full#supplementary-material

## Footnotes

^1^ http://www.r-project.org/
