# Supplementary material for: Distinguishing COVID-19 From Influenza Pneumonia in the Early Stage Through CT Imaging and Clinical Features
Source: Front Microbiol. 2022 May 6;13:847836. doi: 10.3389/fmicb.2022.847836 (PMC9120763; doi:10.3389/fmicb.2022.847836)
Supplement: Supplementary file 1 [file Data_Sheet_1.docx]

**Supplementary Materials**

**Table of Contents：**

[1. CT imaging protocols and acquisition parameters (**Table E1**).................................2](#_Toc37192033)

[2. Definition of CT imaging features (**Table E2** and **Table E3**)...................................4](#_Definition_of_CT)

3. [CT numerical features of COVID-19 and influenza pneumonia (**Table E4**) 10](#_Toc37192036)

[4. CT qualitative features of COVID-19 and influenza pneumonia (**Table E5**) 12](#_Toc37192037)

[5. Literature comparison between COVID-19 and influenza (**Table E6**)....................16](#g)

# CT imaging protocols and acquisition parameters (Table E1)

We selected patient images that were acquired on various models of multi-row spiral CT scans from GE, Scenaria and Philips scanners. Table E1 shows the CT imaging protocols and acquisition parameters of each medical institutions.

**Table E1**: CT imaging protocols and acquisition parameters of each medical institutions

| CT imaging protocols and acquisition parameters | Yongzhou people's hospital, Hunan province | The first affiliated hospital of Shantou university medical college | Shantou central hospital, Guangdong province | Huizhou central hospital, Guangdong province | Meizhou people’s hospital, Guangdong province |
| --- | --- | --- | --- | --- | --- |
| CT scanner | 64-section scanner (SCENARIA 64 CT, Hitachi Medical, Japan). | 64-section scanner (GE CT Discovery 750 HD) | 64-section scanner (Ingenuity CT, PHILIPS) | 64-section scanner (Ingenuity CT, PHILIPS) | 64-slice multidetector spiral CT (Somatom Definition AS, Siemens, Guangdong, China) |
| Tube voltage (kV) | 130 | 120 | 120 | 120 | 120 |
| Automatic tube current (mA) | 180-350 | 320 | 132-459 | 400 -500 | 150 |
| Iterative reconstruction technique | + | + | + | + | + |
| Detector (mm) | 64 | 40 | 64 | 64 | 128 |
| Rotation time (s) | 0.5 | 0.5 | 0.75 | 0.5 | 0.5 |
| Section thickness (mm) | 5 | 5 | 5 | 5 | 1.5 |
| Collimation | 0.6 | 0.6 | 0.625 | 0.625 | 0.6 |
| Pitch | 0.99 | 1.5 | 0.98 | 0.9 | 1.2 |
| Matrix | 512 × 512 | 512 × 512 | 512 × 512 | 512 × 512 | 512 × 512 |

# Definition of CT imaging features (Table E2 and Table E3)

# Table E2 presents definitions of radiological feature, which could benefit to understand each feature in CT images and read smoothly. Part of these definitions are cited from study of Hansell DM, et al in 2008^1^.

**Table E2** Definition of CT numerical imaging features

| CT numerical imaging features | Definition |
| --- | --- |
| Total number of pure GGO | The total number of pure GGO in the bilateral lung |
| Number of pure GGO in peripheral area | The total number of pure GGO in the outer third of the lung |
| Number of pure GGO in central area | The total number of pure GGO in the inner two thirds of the lung |
| Number of pure GGO in both peripheral and central area | The total number of pure GGO in both the outer third and inner two thirds of the lung |
| Total number of mixed GGO | The total number of mixed GGO in the bilateral lung |
| Number of mixed GGO in peripheral area | The total number of mixed GGO in the outer third of the lung |
| Number of mixed GGO in central area | The total number of mixed GGO in the inner two thirds of the lung |
| Number of mixed GGO in both peripheral and central area | The total number of mixed GGO in both the outer third and inner two thirds of the lung |
| Total number of consolidation | The total number of consolidation in the bilateral lung |
| Total number of solid nodules | The total number of solid nodules in the bilateral lung |
| Total number of lesions in peripheral area | The total number of pure GGO and mixed GGO in the outer third of the lung |
| Total number of lesions in central area | The total number of pure GGO and mixed GGO in the inner two thirds of the lung |
| Total number of lesions in both peripheral and central area | The total number of pure GGO and mixed GGO in both inner two thirds and outer third of the lung |
| Lesion sizes < 1cm | The total number of pure GGO and mixed GGO with diameter < 1cm in the lung |
| Lesion sizes: 1cm to 3cm | The total number of pure GGO and mixed GGO with 1cm ≤ diameter ≤3 cm in the lung |
| Lesion sizes : 3cm to half of segments | The total number of pure GGO and mixed GGO with 3< diameter≤ half of segments in the lung |
| Lesion sizes:＞half of segments | The total number of pure GGO and mixed GGO with diameter > half of segments in the lung |
| Total scores of bilateral lung | The scores was combined for all involved lobes in the bilateral lung |
| Total scores of right lung | The scores was combined for all involved lobes in the right lung |
| Total scores of left lung | The scores was combined for all involved lobes in the left lung |
| Total scores of bilateral upper lobes lung | The scores was combined for all involved lobes in the bilateral upper lobes lung |
| Total scores of bilateral lower lobes lung | The scores was combined for all involved lobes in the bilateral lower lobes lung |
| Number of right lung lobes affected | The total number of involved lobes in the right lung |
| Number of left lung lobes affected | The total number of involved lobes in the left lung |
| Total number of bilateral lung involved segments | The total number of involved lobes in the bilateral lung |
| Total number of bilateral upper lobes involved segments | The total number of involved lobes in the bilateral upper lobes |
| Total number of bilateral lower lobes involved segments | The total number of involved lobes in the bilateral lower lobes |

**Table E3**: Definition of CT qualitative imaging features

| CT qualitative imaging features | Definition |
| --- | --- |
| Ground glass opacity(GGO) | It appears as hazy increased opacity of lung, with preservation of bronchial and vascular margins. |
| Pure GGO | Equal to GGO |
| Pure GGO in peripheral area | The pure GGO in the outer third of the lung |
| Pure GGO in central area | The pure GGO in the inner two thirds of the lung |
| Pure GGO in both peripheral and central area | The pure GGO in the both outer third and inner two thirds of the lung |
| Consolidation | Consolidation appears as a homogeneous increase in pulmonary parenchymal attenuation that obscures the margins of vessels and airway walls. |
| Mixed GGO | The combination of GGO and consolidation |
| Mixed GGO in peripheral area | The mixed GGO in the outer third of the lung |
| Mixed GGO in central area | The mixed GGO in the inner two thirds of the lung |
| Mixed GGO both peripheral and central area | The mixed GGO in the both outer third and inner two thirds of the lung |
| Emphysema | The CT appearance of emphysema consists of focal areas or regions of low attenuation, usually without visible walls. |
| Air bronchogram | An air bronchogram is a pattern of air-filled (low-attenuation) bronchi on a back-ground of opaque (high-attenuation) air-less lung. |
| Interlobular septal thickening | On CT scans, disease affecting one of the components of the septa may be responsible for thickening and so render septa visible. |
| Crazy-paving pattern | This pattern appears as thickened interlobular septa and intralobular lines superimposed on a background of ground-glass opacity, resembling irregularly shaped paving stones. |
| Tree-in-bud | The tree-in-bud pattern represents centrilobular branching structures that resemble a budding tree. |
| Cavity | A cavity is a gas-filled space, seen as a lucency or low-attenuation area, within pulmonary consolidation, a mass, or a nodule. |
| Lymphadenopathy | By common usage, the term lymphadenopathy is usually restricted to enlargement, due to any cause, of the lymph nodes. Somewhat arbitrary thresholds for the upper limit of normal of 1 cm in short-axis diameter for mediastinal nodes and 3 mm for most hilar nodes have been reported. |
| Offending vessel augmentation in lesions | The vessel associated with lesions is enlarged in CT image. |
| Overall condition of lesions | The total number of lesion in the lung, including 0,1 and ≥ 2, corresponding to normal, single and multiple. |
| Pleural thickening | The involved pleura is thickening as elevated flat or nodular lesions. |
| Pleural traction | Pleural traction was defined as depression areas of pleural thickening |
| Pleural effusions | The CT appearance of pleural effusions consists of fluid in the pleural space. |
| Bronchial wall thickening | Bronchial wall thickness was defined as bronchovascular interstitial thickening |
| Linear opacities | Linear opacities was defined as continuous thickening of peribronchial area |

|  | Table E4: CT numerical imaging features of COVID-19 and influenza pneumonia patients | | | |  |
| --- | --- | --- | --- | --- | --- |
|  | Imaging features | COVID-19 (*n*=73) | influenza (*n*=48) | *P*^b^ -value |  |
|  | Number of pure GGO |  |  |  |  |
|  | Total | 6.78±11.28 | 2.75±5.33 | 0.010* |  |
|  | Peripheral area | 4.81±7.15 | 1.92±3.16 | 0.003* |  |
|  | Central / both peripheral and central area | 1.90±4.12 | 0.94±3.27 | 0.174 |  |
|  | Number of mixed GGO | |  |  |  |
|  | Total | 6.27±9.52 | 4.67±7.41 | 0.325 |  |
|  | Peripheral area | 4.60±6.92 | 2.15±4.12 | 0.016* |  |
|  | Central / both peripheral and central area | 1.77±3.29 | 2.50±4.03 | 0.275 |  |
|  | Total number of consolidation and solid nodules | | | |  |
|  | Consolidation | 0.60±1.65 | 1.60±2.52 | 0.018* |  |
|  | Solid nodules | 0.12±0.37 | 0.27±0.64 | 0.113 |  |
|  | Total number of lesions |  |  |  |  |
|  | Peripheral area | 10.74±13.69 | 5.15±6.63 | 0.003* |  |
|  | Central area | 1.82±4.32 | 1.90±3.09 | 0.919 |  |
|  | Both peripheral and central area | 2.18±5.40 | 2.56±3.02 | 0.655 |  |
|  | Lesion sizes |  |  |  |  |
|  | < 1cm | 3.11±5.05 | 3.04±3.99 | 0.938 |  |
|  | 1cm to 3cm | 8.29±14.24 | 3.21±4.19 | 0.005* |  |
|  | 3cm to half of segments | 2.40±4.97 | 1.92±2.74 | 0.542 |  |
|  | ＞half of segments | 0.90±2.10 | 1.50±1.94 | 0.118 |  |
|  | Total scores of involved lung zones | |  |  |  |
|  | Bilateral lung | 5.05±4.03 | 6.35±5.35 | 0.155 |  |
|  | Right lung | 2.90±2.45 | 3.31±3.06 | 0.419 |  |
|  | Left lung | 2.15±1.86 | 3.10±2.62 | 0.032* |  |
|  | Bilateral upper lobes | 1.70±1.45 | 2.06±2.37 | 0.345 |  |
|  | Bilateral lower lobes | 2.59±2.18 | 3.69±2.52 | 0.015* |  |
|  | Number of lobes affected |  |  |  |  |
|  | Right lung | 1.88±1.20 | 1.75±1.02 | 0.535 |  |
|  | Left lung | 1.34±0.82 | 1.33±0.72 | 0.950 |  |
|  | Total number of involved lung segments | | | |  |
|  | Bilateral lung | 3.22±1.86 | 3.04±1.61 | 0.589 |  |
|  | Bilateral upper lobes | 1.30±0.88 | 1.00±0.88 | 0.067 |  |
|  | Bilateral lower lobes | 1.40±0.83 | 1.65±0.67 | 0.085 |  |
|  | Note: * Data with statistical significance. Results are mean value with standard deviation. *P*^b^: student’s *t* tes. | | | |  |

|  | Table E5: CT qualitative imaging features of COVID-19 and influenza pneumonia patients | | | |  |
| --- | --- | --- | --- | --- | --- |
|  | Imaging features | COVID-19 (*n*=73) | influenza pneumonia(*n*=48) | *P*-value |  |
|  | Pure GGO | |  | 0.008^a^* |  |
|  | Negative | 18(24.7%) | 23(47.9%) |  |  |
|  | Positive | 55(75.3%) | 25(25.1%) |  |  |
|  | Pure GGO in peripheral area | |  | 0.004^a^* |  |
|  | Negative | 19(26.0%) | 25(52.1%) |  |  |
|  | Positive | 54(74.0%) | 23(47.9%) |  |  |
|  | Pure GGO in central / both peripheral and central area | | | 0.062^a^ |  |
|  | Negative | 41(56.2%) | 35(72.9%) |  |  |
|  | Positive | 32(43.8%) | 13(27.1%) |  |  |
|  | Mixed GGO |  |  | 0.020^a^* |  |
|  | Negative | 16(21.9%) | 20(41.7%) |  |  |
|  | Positive | 57(78.1%) | 28(58.3%) |  |  |
|  | Mixed GGO in peripheral area | | | <0.001^a^* |  |
|  | Negative | 18(24.7%) | 27(56.3%) |  |  |
|  | Positive | 55(75.3%) | 21(43.7%) |  |  |
|  | Mixed GGO in central / both peripheral and central area | | |  |  |
|  | Negative | 39(53.4%) | 26(54.2%) | 0.936^a^ |  |
|  | Positive | 34(46.6%) | 22(45.8%) |  |  |
|  | Consolidation |  |  | <0.001^a^* |  |
|  | Negative | 56(76.7%) | 21(43.8%) |  |  |
|  | Positive | 17(23.3%) | 27(56.2%) |  |  |
|  | Interlobular septal thickening | |  | 0.037^a^* |  |
|  | Negative | 33(45.21%) | 31(64.58%) |  |  |
|  | Positive | 40(54.79%) | 17(35.42%) |  |  |
|  | Crazy paving pattern |  |  | <0.001^a^* |  |
|  | Negative | 35(47.95%) | 41(85.42%) |  |  |
|  | Positive | 38(52.05%) | 7(14.58%) |  |  |
|  | Tree in bud |  |  | 0.978^a^ |  |
|  | Negative | 64(87.67%) | 42(87.50%) |  |  |
|  | Positive | 9(12.33%) | 6(12.50%) |  |  |
|  | Pleural thickening |  |  | 0.086^a^ |  |
|  | Negative | 39(53.42%) | 18(37.50%) |  |  |
|  | Positive | 34(46.58%) | 30(62.50%) |  |  |
|  | Offending vessel augmentation in lesions | |  | 0.021^a^* |  |
|  | Negative | 20(27.40%) | 23(47.92%) |  |  |
|  | Positive | 53(72.60%) | 25(52.08%) |  |  |
|  | Overall condition of lesions | |  | 0.834^c^ |  |
|  | Normal | 9(12.33 %) | 3(6.25 %) |  |  |
|  | Single | 5(6.85 %) | 6(12.50 %) |  |  |
|  | Multiple | 59(80.82 %) | 39(81.25 %) |  |  |
|  | Total number of lobes affected in bilateral lung | | | 0.502^a^ |  |
|  | ＜3 lobes | 26(35.62%) | 20(41.67%) |  |  |
|  | ≥ 3 lobes | 47(64.38%) | 28(58.33%) |  |  |
|  | Pleural traction |  |  | 0.007^a^* |  |
|  | Negative | 38(52.05%) | 13(27.08%) |  |  |
|  | Positive | 35(47.95%) | 35(72.92%) |  |  |
|  | Linear opacities |  |  | 0.248^a^ |  |
|  | Negative | 32(43.84%) | 16(33.33%) |  |  |
|  | Positive | 41(56.16%) | 32(66.67%) |  |  |
|  | Emphysema |  |  | 0.045^a^* |  |
|  | Negative | 67(91.78%) | 38(79.17%) |  |  |
|  | Positive | 6(8.22%) | 10(20.83%) |  |  |
|  | Air bronchogram |  |  | 0.374^a^ |  |
|  | Negative | 32(43.84%) | 25(52.08%) |  |  |
|  | Positive | 41(56.16%) | 23(47.92%) |  |  |
|  | Bronchial wall thickening |  |  | 0.715^a^ |  |
|  | Negative | 31(42.47%) | 22(45.83%) |  |  |
|  | Positive | 42(57.53%) | 26(54.17%) |  |  |
|  | Pleural effusions |  |  | <0.001^a^* |  |
|  | Negative | 73(100.0%) | 38(79.17%) |  |  |
|  | Positive | 0(0.00%) | 10(20.83%) |  |  |
|  | Cavity |  |  | 0.517^a^ |  |
|  | Negative | 71(97.26%) | 48(100.0%) |  |  |
|  | Positive | 2(2.74%) | 0(0.00%) |  |  |
|  | Lymphadenopathy |  |  | 0.047^a^* |  |
|  | Negative | 73(100.0%) | 44(91.67%) |  |  |
|  | Positive | 0(0.0%) | 4(8.33%) |  |  |
|  | Note: * Data with statistical significance. Results are measurements with corresponding ratio in parentheses. *P*^a^: chi square test, *P*^c^: Kruskal-Wallis *H* test. | | | |  |

**Table E6** Literature comparison between COVID-19 and influenza

| Author/year | Method | Sample size  (COVID-19 vs.Influenza Virus) | Results | | |
| --- | --- | --- | --- | --- | --- |
| Zhao et al^2^/2021 | CT and clinical features | 24 vs. 79 | Clinical model:  AUC: 0.819;  Sensitivity:0.783; Specificity: 0.747 | CT model:  AUC: 0.927;  Sensitivity: 0.750;  Specificity: 0.962 | Combined model:  AUC: 0.961;  Sensitivity: 0.87;  Specificity: 0.96 |
| Huang et al^3^/2021 | CT-based radiomics combined with signs | 89 vs. 65 | Radiomics model:  AUC: 0.888;  Sensitivity:0.865; Specificity: 0.784 | CT Signs model:  AUC: 0.906;  Sensitivity: 0.865; Specificity: 0.815 | Combined model:  AUC: 0.959;  Sensitivity: 0.899;  Specificity: 0.907 |
| Shi et al^4^/2021 | LASSO model | 291 vs. 97 | Primary cohort LASSO model:  AUC: 0.963;  Sensitivity: 0.897;  Specificity: 0.897 | | Validation cohort LASSO model:  AUC: 0.943;  Sensitivity: 0.862;  Specificity: 0.897 |
| Present study | CT and clinical features | 73 vs. 48 | Clinical model:  AUC: 0.879;  Sensitivity:0.875; Specificity: 0.739 | CT model:  AUC: 0.956;  Sensitivity:0.875;  Specificity: 0.932 | Combined model:  AUC: 0.991;  Sensitivity: 0.937;  Specificity: 0.958 |

COVID-19, coronavirus disease 2019; AUC, area under the curve; LASSO, least absolute shrinkage and selection operator; CT, computed tomography

Reference

1. Hansell DM, Bankier AA, MacMahon H, McLoud TC, Müller NL, Remy J. Fleischner Society: glossary of terms for thoracic imaging. *Radiology*. Mar 2008;246(3):697-722. doi:10.1148/radiol.2462070712

2. Zhao S, Huang Z, Zeng H, et al. Combining initial chest CT with clinical variables in differentiating coronavirus disease 2019 (COVID-19) pneumonia from influenza pneumonia. *Scientific reports*. Mar 19 2021;11(1):6422. doi:10.1038/s41598-021-85779-1

3. Huang Y, Zhang Z, Liu S, et al. CT-based radiomics combined with signs: a valuable tool to help radiologist discriminate COVID-19 and influenza pneumonia. *BMC medical imaging*. Feb 17 2021;21(1):31. doi:10.1186/s12880-021-00564-w

4. Shi WY, Hu SP, Zhang HL, et al. Differential Diagnosis of COVID-19 Pneumonia From Influenza A (H1N1) Pneumonia Using a Model Based on Clinicoradiologic Features. *Frontiers in medicine*. 2021;8:651556. doi:10.3389/fmed.2021.651556
